# Broncho-pneumopathies à répétition: penser à un syndrome de Kartagener

**DOI:** 10.11604/pamj.2015.22.38.7877

**Published:** 2015-09-17

**Authors:** Madiha Mahfoudhi, Khaled Khamassi

**Affiliations:** 1 Service de Médecine Interne A, Hôpital Charles Nicolle, Tunis, Tunisie; 2 Service ORL, Hôpital Charles Nicolle, Tunis, Tunisie

**Keywords:** Broncho-pneumopathies, syndrome de Kartagener, polypectomie, bronchopneumopathies, Kartagener syndrome, polypectomy

## Image en medicine

Le syndrome de Kartagener est une affection héréditaire rare de transmission autosomique récessive. Il se caractérise par une triade: bronchectasies, rhinosinusite chronique et situs inversus. L’évolution est dominée par la survenue d'infections respiratoires haute et basses. Patiente âgée de 21 ans, admise pour obstruction nasale bilatérale, hyposmie et rhinorrhée évoluant depuis 3 ans. Elle présentait depuis le jeune âge des broncho-pneumopathies à répétition non explorées. L'examen physique a retrouvé des râles bronchiques bilatéraux, des bruits de cœur plus bien perçus du côté droit, une rhinorrhée purulente avec muqueuse nasale congestive, des cornets moyens hypertrophiés et des polypes translucides comblant les 2 méats moyens. L'otoscopie a objectivé des tympans complets rétractés. Le bilan biologique a révélé une anémie hypochrome microcytaire. L'audiométrie tonale a montré une surdité de transmission bilatérale à 40 dB. A l'impédancemétrie, les courbes étaient déviées vers les pressions négatives et les réflexes stapédiens étaient absents. La radiographie du thorax a montré une dextrocardie. La TDM du massif facial et thoracique a montré un comblement des sinus maxillaires, des cellules ethmoïdales, et un situs inversus sans anomalies des gros vaisseaux. L’échographie abdominale n'a pas montré de transposition de viscères. Le traitement médical s'est basé sur la prescription d'antibiotiques et de corticoïdes par voie locale et générale, associée à une kinésithérapie respiratoire. Le traitement chirurgical a consisté en une polypectomie, méatotomie moyenne bilatérale et mise en place de 2 drains trans-tympaniques. L’évolution était marquée par une nette amélioration clinique avec un recul de 7 ans.

**Figure 1:**
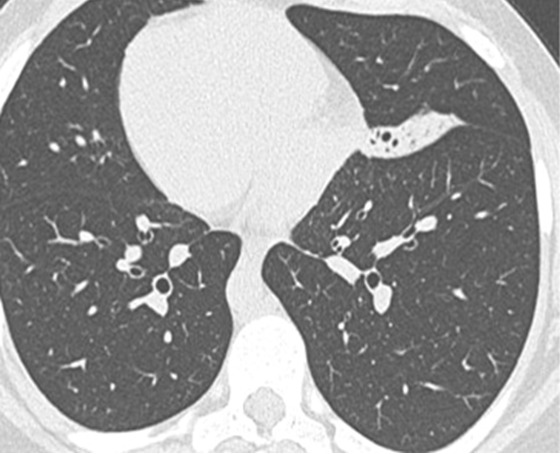
TDM thoracique: dextrocardie (situs inversus)

